# A polymorph of 2,4-dinitro­phenyl­hydrazine

**DOI:** 10.1107/S1600536813004571

**Published:** 2013-02-23

**Authors:** Kiichi Amimoto, Hiromitsu Nishiguchi

**Affiliations:** a Department of Science Education, Graduate School of Education, Hiroshima University, 1-1-1 Kagamiyama, Higashi-Hiroshima, Hiroshima, Japan

## Abstract

The crystal structure of a previously unreported polymorph (form II) of 2,4-dinitro­phenyl­hydrazine (DNPH), C_6_H_6_N_4_O_4_, was determined at 90 K. The first polymorph (form I) is described in the monoclinic space group *P*2_1_/*c* [Okabe *et al.* (1993 ▶). *Acta Cryst.* C**49**, 1678–1680; Wardell *et al.* (2006 ▶). *Acta Cryst.* C**62**, o318–320], whereas form II is in the monoclinic space group *Cc*. The mol­ecular structures in forms I and II are closely similar, with the nitro groups at the 2- and 4-positions being almost coplanar with the benzene ring [dihedral angles of 3.54 (1) and 3.38 (1)°, respectively in II]. However, their packing arrangements are completely different. Form I exhibits a herringbone packing motif, whereas form II displays a coplanar chain structure. Each chain in form II is connected to adjacent chains by the inter­molecular inter­action between hydrazine NH_2_ and 2-nitro groups, forming a sheet normal to (101). The sheet is stabilized by N—H⋯π inter­actions.

## Related literature

For the use of DNPH for the identification of a carbonyl group, see: Brady & Elsmie (1926 ▶); Williamson *et al.* (2006 ▶). For the crystal structure of the first polymorph of DNPH, see: Okabe *et al.* (1993 ▶); Wardell *et al.* (2006 ▶).
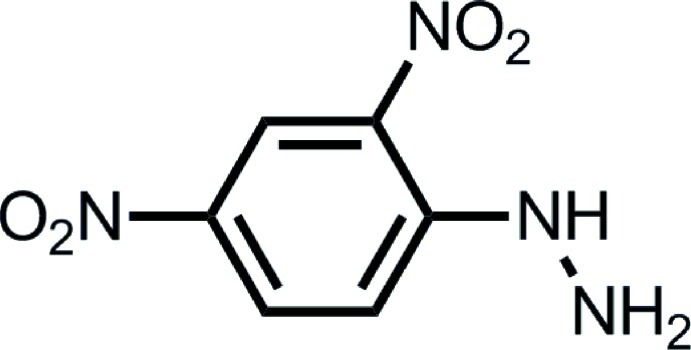


## Experimental

### 

#### Crystal data

C_6_H_6_N_4_O_4_
*M* *_r_* = 198.15
Monoclinic, 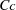

*a* = 12.697 (5) Å
*b* = 9.179 (5) Å
*c* = 7.662 (5) Å
β = 123.315 (5)°
*V* = 746.2 (7) Å^3^
*Z* = 4
Mo *K*α radiation
μ = 0.15 mm^−1^
*T* = 90 K
0.3 × 0.2 × 0.15 mm

#### Data collection

Bruker SMART APEX CCD area-detector diffractometer
1878 measured reflections
1433 independent reflections
1424 reflections with *I* > 2σ(*I*)
*R*_int_ = 0.019

#### Refinement

*R*[*F* ^2^ > 2σ(*F* ^2^)] = 0.027
*wR*(*F* ^2^) = 0.072
*S* = 1.06
1433 reflections
151 parameters
2 restraints
All H-atom parameters refined
Δρ_max_ = 0.25 e Å^−3^
Δρ_min_ = −0.17 e Å^−3^

### 

Data collection: *APEX2* (Bruker, 2009 ▶); cell refinement: *SAINT* (Bruker, 2009 ▶); data reduction: *SAINT*; program(s) used to solve structure: *SIR92* (Altomare *et al.*, 1994 ▶); program(s) used to refine structure: *SHELXL97* (Sheldrick, 2008 ▶); molecular graphics: *Mercury* (Macrae *et al.*, 2008 ▶) and *ORTEP-3 for Windows* (Farrugia, 2012 ▶); software used to prepare material for publication: *WinGX* (Farrugia, 2012 ▶) and *publCIF* (Westrip, 2010 ▶).

## Supplementary Material

Crystal structure: contains datablock(s) global, I. DOI: 10.1107/S1600536813004571/ds2226sup1.cif

Structure factors: contains datablock(s) I. DOI: 10.1107/S1600536813004571/ds2226Isup2.hkl

Supplementary material file. DOI: 10.1107/S1600536813004571/ds2226Isup3.cml

Additional supplementary materials:  crystallographic information; 3D view; checkCIF report

## Figures and Tables

**Table 1:** Hydrogen-bond geometry (Å, °) *Cg* is the centroid of the C1–C6 ring.

*D*—H⋯*A*	*D*—H	H⋯*A*	*D*⋯*A*	*D*—H⋯*A*
N3—H3*N*⋯O4^i^	0.81 (3)	2.47 (3)	2.919 (3)	116 (2)
N4—H4*NA*⋯O1^ii^	0.90 (3)	2.43 (3)	3.215 (3)	145.1 (17)
N4—H4*NA*⋯O3^iii^	0.90 (3)	2.35 (3)	3.052 (3)	135.0 (15)
N4—H4*NB*⋯O4^i^	1.01 (3)	2.31 (3)	2.981 (3)	123 (2)
N4—H4*NB*⋯O2^iv^	1.01 (3)	2.34 (3)	3.163 (3)	138 (3)
N4—H4*NB*⋯*Cg* ^v^	1.01 (3)	2.91 (4)	3.306 (3)	104 (2)

Symmetry codes: (i) 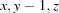; (ii) 
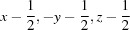
; (iii) 
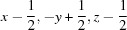
; (iv) 
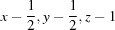
; (v) 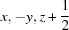.

**Fig. 1.**
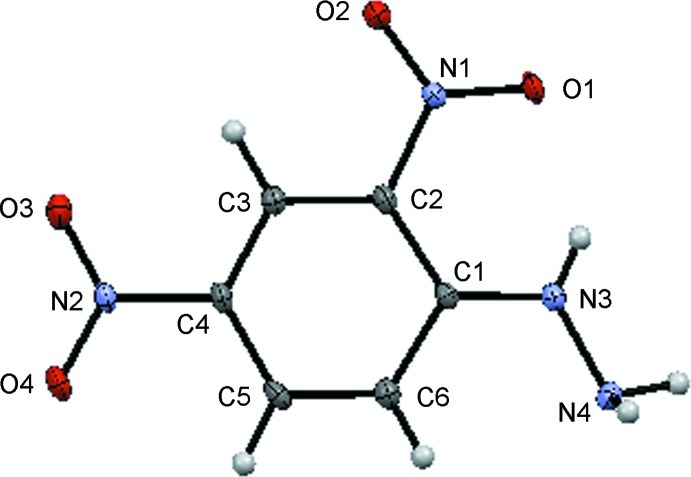
The molecular structure of the title polymorph II, showing the atom-labelling scheme and displacement ellipsoids at the 50% probability level.

**Fig. 2.**
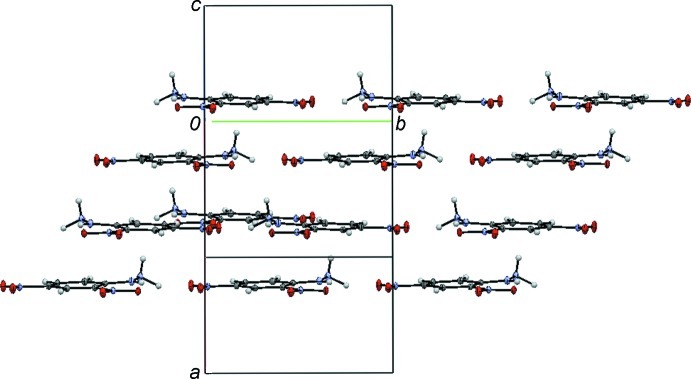
The crystal packing of the title polymorph II showing the 2-D sheet arrangement.

## supplementary crystallographic information

### Comment

The nature and reactivity of carbonyl group is one of the most important topics in organic chemistry. 2,4-Dinitrophenylhydrazine (DNPH) is often used as qualitative test for carbonyl groups in the field of chemical education (Brady & Elsmie 1926; Williamson *et al.*, 2006). DNPH also produces the 2,4-dinitrophenylhydrazone derivatives, which offer a variety of functional organic dye crystals. The crystal structure (I) of DNPH at room temperature and 120 K were reported (Okabe *et al.* 1993; Wardell *et al.*, 2006). In the course of our studies on the development of teaching materials for organic chemistry and novel crystalline materials of organic dyes, we have found the new polymorph (II) of DNPH. The molecular structure in II is almost the same to that in I. The molecular structure in II adapts the planar conformation: the dihedral angles of nitro groups at the 2- and 4-positions to the benzene ring are 3.54 (1)° and 3.38 (1)°, respectively. In both I and II, there is an intermolecular interaction between hydrazine NH_2_ and 4-nitro group, forming a chain structure. The distinguished difference between I and II originates their molecular arrangements in the chain structure. In I a benzene ring is inclined at 54.86 ° to the adjacent one, forming a herringbone packing motif. On the other hand, all benzene rings on a chain structure in II lie on the same plane. The interatomic distances in II between hydrazine moiety and 4-nitro group are N(3)—O(4) = 2.919 (3) Å and N(4)—O(4) = 2.981 (3) Å, respectively. Each chain is connected to adjacent ones in the same direction by the additional interaction between hydrazine NH_2_ and 2-nitro group, forming a 2-D sheet normal to [1 0 1] plane [N(4)—O(2) = 3.163 (3) Å]. And the 2-D sheets are built up by the offset stacking. The face-to-face stacking of 3.306 (3) Å between centroid of benzene rings and hydrazine N(4) indicates the existence of π–NH_2_ interaction between electron-deficient aromatic ring connected to electron-withdrawing nitro group and electron-donating hydrazine moiety.

### Experimental

Crystals of title polymorph II were obtained by slow evaporation with commercially available DNPH using 1,4-dioxane as solvent.

### Refinement

All hydrogen atoms were found in a difference Fourier map and refined isotropically.

### Crystal data

**Table:**

C_6_H_6_N_4_O_4_	*F*(000) = 408
*M**_r_* = 198.15	*D*_x_ = 1.764 Mg m^−^^3^
Monoclinic, *C**c*	Mo *K*α radiation, λ = 0.71069 Å
Hall symbol: C -2yc	Cell parameters from 1986 reflections
*a* = 12.697 (5) Å	θ = 2.9–28.8°
*b* = 9.179 (5) Å	µ = 0.15 mm^−^^1^
*c* = 7.662 (5) Å	*T* = 90 K
β = 123.315 (5)°	Block, red
*V* = 746.2 (7) Å^3^	0.3 × 0.2 × 0.15 mm
*Z* = 4	

### Data collection

**Table:**

Bruker SMART APEX CCD area-detector diffractometer	1424 reflections with *I* > 2σ(*I*)
Radiation source: fine-focus sealed tube	*R*_int_ = 0.019
Graphite monochromator	θ_max_ = 28.9°, θ_min_ = 2.9°
Detector resolution: 8.333 pixels mm^-1^	*h* = −17→10
phi and ω scan	*k* = −10→11
1878 measured reflections	*l* = −9→9
1433 independent reflections	

### Refinement

**Table:**

Refinement on *F*^2^	Primary atom site location: structure-invariant direct methods
Least-squares matrix: full	Secondary atom site location: difference Fourier map
*R*[*F*^2^ > 2σ(*F*^2^)] = 0.027	Hydrogen site location: inferred from neighbouring sites
*wR*(*F*^2^) = 0.072	All H-atom parameters refined
*S* = 1.06	*w* = 1/[σ^2^(*F*_o_^2^) + (0.0446*P*)^2^ + 0.1782*P*] where *P* = (*F*_o_^2^ + 2*F*_c_^2^)/3
1433 reflections	(Δ/σ)_max_ < 0.001
151 parameters	Δρ_max_ = 0.25 e Å^−^^3^
2 restraints	Δρ_min_ = −0.17 e Å^−^^3^

### Special details

**Table:**

Geometry. All e.s.d.'s (except the e.s.d. in the dihedral angle between two l.s. planes) are estimated using the full covariance matrix. The cell e.s.d.'s are taken into account individually in the estimation of e.s.d.'s in distances, angles and torsion angles; correlations between e.s.d.'s in cell parameters are only used when they are defined by crystal symmetry. An approximate (isotropic) treatment of cell e.s.d.'s is used for estimating e.s.d.'s involving l.s. planes.
Refinement. Refinement of *F*^2^ against ALL reflections. The weighted *R*-factor *wR* and goodness of fit *S* are based on *F*^2^, conventional *R*-factors *R* are based on *F*, with *F* set to zero for negative *F*^2^. The threshold expression of *F*^2^ > σ(*F*^2^) is used only for calculating *R*-factors(gt) *etc*. and is not relevant to the choice of reflections for refinement. *R*-factors based on *F*^2^ are statistically about twice as large as those based on *F*, and *R*- factors based on ALL data will be even larger.

### Fractional atomic coordinates and isotropic or equivalent isotropic displacement parameters (Å^2^)

**Table:**

	*x*	*y*	*z*	*U*_iso_*/*U*_eq_	
C1	0.22967 (12)	0.03156 (15)	0.6894 (2)	0.0099 (3)	
C2	0.33423 (13)	0.08580 (17)	0.8822 (2)	0.0111 (3)	
C3	0.35605 (13)	0.23444 (16)	0.9232 (2)	0.0113 (3)	
C4	0.27641 (14)	0.33162 (17)	0.7701 (2)	0.0121 (3)	
C5	0.17463 (14)	0.28510 (15)	0.5739 (2)	0.0126 (3)	
C6	0.15142 (14)	0.13877 (16)	0.5363 (2)	0.0126 (3)	
N1	0.42299 (11)	−0.00982 (14)	1.04810 (18)	0.0107 (2)	
N2	0.30055 (12)	0.48633 (14)	0.8159 (2)	0.0127 (3)	
N3	0.20280 (12)	−0.11032 (13)	0.6487 (2)	0.0125 (2)	
N4	0.09528 (12)	−0.15491 (14)	0.4547 (2)	0.0145 (3)	
O1	0.41196 (11)	−0.14375 (11)	1.01911 (17)	0.0148 (2)	
O2	0.50714 (11)	0.04357 (13)	1.21641 (18)	0.0167 (2)	
O3	0.38720 (12)	0.52367 (12)	0.99101 (19)	0.0191 (3)	
O4	0.23265 (12)	0.57352 (12)	0.67622 (19)	0.0212 (3)	
H3	0.431 (2)	0.268 (2)	1.060 (3)	0.008 (4)*	
H3N	0.252 (3)	−0.162 (3)	0.746 (4)	0.019 (5)*	
H5	0.118 (2)	0.350 (2)	0.464 (4)	0.020 (5)*	
H4NA	0.033 (2)	−0.171 (2)	0.476 (4)	0.028 (5)*	
H4NB	0.116 (3)	−0.253 (3)	0.422 (5)	0.037 (7)*	
H6	0.082 (2)	0.110 (3)	0.414 (4)	0.020 (5)*	

### Atomic displacement parameters (Å^2^)

**Table:**

	*U*^11^	*U*^22^	*U*^33^	*U*^12^	*U*^13^	*U*^23^
C1	0.0112 (7)	0.0085 (7)	0.0118 (7)	0.0013 (4)	0.0074 (6)	0.0009 (5)
C2	0.0126 (7)	0.0085 (7)	0.0138 (6)	0.0015 (5)	0.0082 (6)	0.0013 (5)
C3	0.0122 (7)	0.0096 (6)	0.0133 (7)	0.0002 (5)	0.0078 (6)	0.0002 (5)
C4	0.0144 (8)	0.0070 (7)	0.0169 (7)	0.0009 (5)	0.0100 (6)	0.0011 (5)
C5	0.0147 (7)	0.0090 (6)	0.0149 (7)	0.0041 (5)	0.0087 (6)	0.0045 (5)
C6	0.0139 (7)	0.0103 (7)	0.0147 (6)	0.0005 (5)	0.0085 (6)	0.0005 (5)
N1	0.0114 (6)	0.0086 (5)	0.0111 (6)	0.0013 (4)	0.0057 (5)	0.0016 (4)
N2	0.0137 (6)	0.0072 (5)	0.0178 (6)	0.0008 (5)	0.0089 (5)	0.0008 (5)
N3	0.0138 (6)	0.0086 (5)	0.0134 (6)	0.0000 (5)	0.0064 (5)	0.0005 (5)
N4	0.0137 (6)	0.0108 (5)	0.0141 (6)	−0.0013 (4)	0.0047 (5)	−0.0028 (4)
O1	0.0171 (5)	0.0067 (4)	0.0201 (6)	0.0020 (4)	0.0099 (5)	0.0019 (4)
O2	0.0174 (5)	0.0111 (5)	0.0149 (5)	0.0001 (4)	0.0046 (4)	0.0012 (4)
O3	0.0208 (6)	0.0101 (6)	0.0228 (7)	−0.0019 (4)	0.0098 (5)	−0.0013 (4)
O4	0.0255 (7)	0.0078 (5)	0.0233 (7)	0.0026 (4)	0.0089 (6)	0.0033 (4)

### Geometric parameters (Å, º)

**Table:**

C1—N3	1.3389 (18)	C5—H5	0.96 (2)
C1—C2	1.428 (2)	C6—H6	0.90 (2)
C1—C6	1.4320 (19)	N1—O2	1.2374 (17)
C2—C3	1.393 (2)	N1—O1	1.2434 (17)
C2—N1	1.4430 (19)	N2—O3	1.2273 (19)
C3—C4	1.3753 (19)	N2—O4	1.2312 (18)
C3—H3	1.01 (2)	N3—N4	1.4181 (18)
C4—C5	1.407 (2)	N3—H3N	0.81 (3)
C4—N2	1.4545 (18)	N4—H4NA	0.91 (2)
C5—C6	1.371 (2)	N4—H4NB	1.01 (3)
			
N3—C1—C2	123.59 (13)	C5—C6—C1	121.93 (14)
N3—C1—C6	120.28 (13)	C5—C6—H6	118.7 (15)
C2—C1—C6	116.12 (13)	C1—C6—H6	119.3 (15)
C3—C2—C1	122.10 (13)	O2—N1—O1	121.72 (12)
C3—C2—N1	115.77 (13)	O2—N1—C2	119.11 (13)
C1—C2—N1	122.12 (13)	O1—N1—C2	119.16 (12)
C4—C3—C2	118.74 (14)	O3—N2—O4	123.23 (13)
C4—C3—H3	121.4 (12)	O3—N2—C4	118.69 (11)
C2—C3—H3	119.8 (12)	O4—N2—C4	118.08 (12)
C3—C4—C5	121.90 (14)	C1—N3—N4	120.02 (12)
C3—C4—N2	117.94 (13)	C1—N3—H3N	113.0 (17)
C5—C4—N2	120.16 (12)	N4—N3—H3N	126.9 (17)
C6—C5—C4	119.14 (13)	N3—N4—H4NA	106.8 (15)
C6—C5—H5	117.0 (14)	N3—N4—H4NB	106.4 (17)
C4—C5—H5	123.8 (14)	H4NA—N4—H4NB	106 (2)

### Hydrogen-bond geometry (Å, º)

Cg is the centroid of the C1–C6 ring.

**Table:**

*D*—H···*A*	*D*—H	H···*A*	*D*···*A*	*D*—H···*A*
N3—H3*N*···O4^i^	0.81 (3)	2.47 (3)	2.919 (3)	116 (2)
N4—H4*NA*···O1^ii^	0.90 (3)	2.43 (3)	3.215 (3)	145.1 (17)
N4—H4*NA*···O3^iii^	0.90 (3)	2.35 (3)	3.052 (3)	135.0 (15)
N4—H4*NB*···O4^i^	1.01 (3)	2.31 (3)	2.981 (3)	123 (2)
N4—H4*NB*···O2^iv^	1.01 (3)	2.34 (3)	3.163 (3)	138 (3)
N4—H4*NB*···*Cg*^v^	1.01 (3)	2.91 (4)	3.306 (3)	104 (2)

Symmetry codes: (i) *x*, *y*−1, *z*; (ii) *x*−1/2, −*y*−1/2, *z*−1/2; (iii) *x*−1/2, −*y*+1/2, *z*−1/2; (iv) *x*−1/2, *y*−1/2, *z*−1; (v) *x*, −*y*, *z*+1/2.
